# Assessing the role of syringe dispensing machines and mobile van outlets in reaching hard-to-reach and high-risk groups of injecting drug users (IDUs): a review

**DOI:** 10.1186/1477-7517-4-14

**Published:** 2007-10-24

**Authors:** Md Mofizul Islam, Katherine M Conigrave

**Affiliations:** 1 STIRC, the University of Sydney, Sydney, NSW, Australia; 2 Drug Health Services, Royal Prince Alfred Hospital, Sydney, NSW, Australia; 3 Faculty of Medicine, the University of Sydney, Sydney, NSW, Australia

## Abstract

Reaching hard-to-reach and high-risk injecting drug users (IDUs) is one of the most important challenges for contemporary needle syringe programs (NSPs). The aim of this review is to examine, based upon the available international experience, the effectiveness of syringe vending machines and mobile van/bus based NSPs in making services more accessible to these hard-to-reach and high-risk groups of IDUs. A literature search revealed 40 papers/reports, of which 18 were on dispensing machines (including vending and exchange machines) and 22 on mobile vans. The findings demonstrate that syringe dispensing machines and mobile vans are promising modalities of NSPs, which can make services more accessible to the target group and in particular to the harder-to-reach and higher-risk groups of IDUs. Their anonymous and confidential approaches make services attractive, accessible and acceptable to these groups. These two outlets were found to be complementary to each other and to other modes of NSPs. Services through dispensing machines and mobile vans in strategically important sites are crucial elements in continuing efforts in reducing the spread of HIV and other blood borne viruses among IDUs.

## Introduction

HIV transmission associated with sharing of contaminated injecting equipment is now a global problem, with more than 110 countries having reported HIV transmission in this context [1]. World Health Organisation (WHO) estimates approximately 10% of all new HIV infections globally can be attributed to the sharing of contaminated injecting equipment [2]. In many parts of the world injecting drug users (IDUs) comprise a far higher proportion of new HIV infections, for example 72% of new HIV infections in Ukraine [3]. An injecting drug user infected with HIV can cause a cascade of new infections in other individuals, not only through sharing of contaminated injecting equipment but also through sexual and perinatal transmission. Hepatitis C and B, two other blood-borne viruses, are far more easily transmitted by blood-blood contact than HIV [4,5] and carry the risk of cirrhosis.

Having experienced the limited outcomes of efforts to significantly eliminate supply and demand for illicit drugs by law enforcement, and in the face of rising prevalence of HIV and other blood-borne viruses, there has been a growing urgency to implement more effective prevention responses to prevent transmission of blood-borne viruses among IDUs. Therefore, authorities have adopted a more realistic approach to drug policy, termed as 'Harm Reduction' or 'Harm minimisation' in which reduction of adverse consequences of drug use is valued as high priority at least as important as reducing demand and supply. The Needle Syringe Program (NSP) or Needle Exchange Program (NEP) is a fundamental component of harm reduction that supports access to sterile injecting equipment for IDUs and discourages sharing of used injecting equipment. Preventive measures through NSP will remain the most effective tool available to reduce the spread of HIV among and from IDUs until an effective and widely deployed vaccine is available.

NSP disease prevention efforts are dependent in part on their ability to attract and maintain contact with IDUs so that injecting equipment, and also education and referrals can be provided. However, IDUs often avoid service providers until a crisis emerges, because they perceive interactions in service settings can be embarrassing, degrading, unhelpful and can be offered at locations and times incompatible with their lives [6]. To overcome these difficulties, a variety of measures have been developed to improve access to and utilization of sterile injecting equipment, and to increase choice for users. These include several methods for distribution, sale or exchange of injecting equipment such as conventional NSPs (housed in a fixed location where IDUs are attended by health staff), pharmacy-based distribution or exchange, dispensing machines (that either sell injecting equipment, provide it for free or in exchange for used equipment) and outreach programmes – often using a mobile van or bus and sometimes through home-visits. In addition, health education and safer injecting advice has been provided. Services through dispensing machines and mobile vans have been reported to be responsive to a wider range of IDUs and most importantly to hidden and harder-to-reach IDUs in the community, who for several reasons do not or cannot attend conventional NSPs [7,8]. The aim of this review is to examine the available evidence for the effectiveness of syringe dispensing machines and mobile van or bus based NSPs in making services accessible to hard-to-reach and high-risk groups of IDUs.

## Methods

Journal publications, conference presentations and proceedings, evaluation reports, and other relevant organizational reports relating to supply of sterile injecting equipment through dispensing machines and/or mobile vans were identified by a comprehensive search of electronic databases such as Medline, Medscape, Current Contents, HealthSTAR, CAB Abstracts, Aidsline, Sociological Abstracts and CINAHL. In addition, experts involved with development and evaluation of current programs or policy were contacted for official reports, policy documents or unpublished materials. In total, 40 papers/reports were found that primarily focused on dispensing machines and/or mobile vans, of which 18 focused on dispensing machines and 22 on mobile vans.

## Results

### Introduction of dispensing machines and mobile vans to NSP

Syringe dispensing machines were first introduced in Copenhagen, Denmark in June 1987 [9] then a few months later in Larvik, Norway. Subsequently they were introduced in several European countries including Switzerland, Germany, France, Italy, the Netherlands, Austria, and also in Australia and New Zealand. These are automatic commercial dispensing machines that exchange new for used syringes, or provide sterile equipment for a coin or free-of-cost. These machines are also known as 'syringe exchange machines', 'syringe vending machines', 'syringe automat' or 'FITPACK^® ^vending machines' (in Australia), 'electronic dispenser', 'distribox^®^' or sometimes simply 'slot machines'. New Zealand introduced a mobile dispensing machine which is wheeled to a front doorway and locked there. This design enables exchange services to continue after hours in a safer way [10].

The NSP-mobile van was first introduced one year earlier, in 1986, in Amsterdam, the Netherlands. It was, in fact, a methadone dispensing bus that also offered syringe and needle exchange [11]. In the same year health workers in London and Liverpool started to exchange needles and syringes using mobile vans [12]. In some settings this form of NSP is known as a 'roving van' or 'mobile bus' or simply 'mobile outlet'. Mobile vans have received much more acceptance than dispensing machines and have been introduced more widely.

### The rationale for dispensing machines and mobile vans in NSPs

Each of these approaches offers the potential to provide injecting equipment to hard-to-reach and high-risk groups of IDUs. For example, some IDUs are concerned to remain anonymous and fear that they may be identified if they try to access sterile injecting equipment from pharmacies or conventional NSPs [13-15]. Many IDUs need access to services in the evening, at night or in weekends. These users strongly feel the need for a non-contact and out of business hours service and consequently dispensing machines were introduced to supply sterile needles and syringes together with condoms, health information pamphlets and other minor health supplies [16]. Dodding & Gaughwin [17] reported that one of the main reasons identified by Australian IDUs for sharing injecting equipment was the relative unavailability of sterile injecting equipment, particularly outside the operating times of pharmacies and conventional NSPs. If attempts are made to continue conventional NSPs at nights and weekends, staffing may be difficult or expensive and also staff may see the work as risky. Dispensing machines overcome these staffing problems. The possibility that judgmental attitudes of some pharmacy staff and NSP staff might discourage some IDUs from obtaining sterile injecting equipment [18,19] was another consideration.

Unlike dispensing machines, mobile vans do not provide completely anonymous access to sterile injecting equipment, but peer staffed mobile vans can render a congenial environment that provides near anonymous access. Mobile vans can cover a greater geographic area and can more readily accommodate changes in local conditions. A van of this sort generally follows a relatively consistent route, and parks at a predictable location at a predictable time, although it can change in response to immediate neighbourhoods' conditions (e.g., increased police presence) or to incorporate additional populations of injecting drug users. One van may visit multiple sites in a single outing. It can provide the benefits of both a fixed and a mobile site. In addition, it can also provide shelter and some security for staff, some privacy for clients, and a consistent service while covering a large geographic area. A roving site also keeps staff members and clients relatively inconspicuous to neighbours, local business people, and police officers.

### Reaching hidden and hard-to-reach IDUs

A subgroup of IDUs are largely marginalised, isolated and socially excluded and highly mobile[54]. They are often not in contact with any services, as they are either unaware of them or do not wish to access them. Corr [54] characterised these groups as drug users who are mostly homeless, female, younger, chaotic and from an ethnic minority. Prisoner IDUs form another important high-risk group. Beginner-IDUs are also hard-to-reach and usually do not define themselves as drug addicts and do not approach NSPs or drug treatment units. None of these groups are mutually exclusive and when these characteristics are multiplied in the same individual, the person is likely to suffer increasing marginalisation [55]. They are highly susceptible to potential adverse health outcomes, particularly blood-borne virus infections as their risky behaviours often go unrecognized. Of the studies/reports reviewed, 37 presented data/results on the ability of dispensing machines and/or mobile vans to reach hidden and hard-to-reach IDUs (Table 1).

**Table 1:** Ability of NSP-mobile vans and dispensing machines to reach high-risk and/or hidden IDUs.

**Syringe dispensing machines**			**Mobile vans**		
**Ref.**	**Location**	**Reached high-risk and/or hidden IDUs**	**Ref.**	**Location**	**Reached high-risk and/or hidden IDUs**

[20]	Vestfold, **Norway**	Yes	[21]	Paris, **France**	Yes
[22]	Rotterdam, the **Netherlands**	Yes	[23]	Paris, **France**	Yes
[24]	Milan, **Italy**	Yes	[25]	Geneva, **Switzerland**	NM
[26]	Sydney, **Australia**	Yes	[27]	Rome, **Italy**	Yes
[28]	Berlin, **Germany**	Yes	[29]	**Spain**	Yes
[30]	Berlin, **Germany**	Yes	[31]	Rockville, **USA**	Yes
[32]	New South Wales, **Australia**	Yes	[33]	Vancouver, **Canada**	Yes
[17]	Perth and Adelaide, **Australia**	Yes, it will*	[34]	Wisconsin, **USA**	Yes
[35]	Hindelbank, **Switzerland**	Yes	[36]	Baltimore, **USA**	Yes
[37]	Realta, **Switzerland**	Yes	[38]	Sicily, **Italy**	Yes
[39]	Marseille, **France**	Yes	[8]	Baltimore, **USA**	Yes
[40]	Vechta, **Germany**	Yes	[41]	St. Petersburg, **Russia**	Yes
[42]	Kalgoorlie, **Australia**	Yes	[43]	Oslo, **Norway**	Yes
[9]	Bremen, **Germany**	NM	[44]	Sofia, **Bulgaria**	Yes
[45]	Marseille, **France**	Yes	[46]	Vancouver, **Canada**	Yes
[47]	Lichtenberg, Lehrter Strasse, **Germany**	Yes	[48]	Madrid, Valencia and some other places of **Spain**	Yes
[49]	Hamburg-Vierlande, **Germany**	NM	[50]	Vilnius, **Lithuania**	Yes
			[51]	New Heaven, CT, **USA**	Yes
[52]	Canberra, **Australia**	Yes	[53]	Melbourne, **Australia**	Yes

* Perception of focus group IDUs and health staff. NM = Not mentioned

#### Dispensing machines

Few studies have attempted to evaluate whether dispensing machines attract hidden, hard-to-reach or high-risk IDUs. Perhaps the most comprehensive studies were performed in Marseille, France where it was found that primary users of vending machines were significantly younger and less likely to live in a house they personally owned or rented; they were also less likely to have been in opioid maintenance treatment [39]. The researchers concluded that the machine outlet seemed to effectively attract a relatively hidden [7] and high-risk segment of IDUs who are less likely to be reached by other programs [39]. In the same city Moatti et al. [45] reported that users of vending machines were younger than those who accessed pharmacy or NSP for needle-syringe and tended to have a lower socioeconomic status. They were significantly less likely than pharmacy users to have a regular job, and more likely than NSP users to be without any resources. Stark et al. [30], in their vending machine study in Berlin, found that machines users were more likely to report a shorter history of injection. The authors explained that early in their injection career IDUs may prefer to obtain injecting equipment anonymously from vending machines. They may not yet consider themselves drug dependent, and may not be willing to contact staffed agencies. This explanation was supported by their findings that only 33% of the IDUs reported current contacts with counselling units. This rate was significantly lower in those with a shorter history of injecting. Similarly Leicht [28] reported that novice IDUs are the main users of machines with most having no contact with other helping agencies for IDUs'. Based upon the findings of a study in Italy, Agnoletto et al [24] underscored the need for complementary use of both exchange machines and mobile vans to modify risk behaviours of drug users who are not in contact with health services.

Evaluation of dispensing machines in Norway showed that these were a successful method of providing sterile equipment to a group until then difficult to reach [20]. Comparable results had also been observed from all the studies in Australia. Dodding & Gaughwin [17] reported that because of the small populations in rural towns, the confidentiality of IDUs in those areas can be particularly important and vending machines may be a valuable form of NSP. Most participants (IDUs and health workers) in this study believed that some IDUs in these areas do not use their local NSPs or pharmacies because of concern for anonymity.

Prisoner-IDUs are at very high risk of blood-borne infections. A range of interlinking factors compound this risk – the large number of IDUs, scarcity of sterile injecting equipment and correspondingly higher prevalence of needle-syringe sharing, rapid turnover of prison populations and hence far more changes in injecting partners [56]. Syringe exchange machines were found to be very effective in increasing access to sterile injecting equipment in prisons in Switzerland and Germany. Their easier and round-the-clock access, high-degree of anonymity; better acceptance by inmates and better control of syringe disposal (one-for-one exchange) made these machines a useful mode of syringe exchange. The availability of injecting equipment through dispensing machines did not lead to an increase in drug use or injection frequency and syringe sharing reduced significantly [47]. Stöver & Jacob [40] reported that anonymous access through exchange machines in a Women's Prison made it more acceptable to the inmates than manual distribution in a Men's Prison. The authors concluded that the level of acceptance among prisoners largely depended on whether anonymity is maintained during needle exchange. However, unfortunately with political changes, all but one syringe exchange machine in prisons in Germany have been removed. Only Lichtenberg-Berlin still offers syringes.

#### Mobile vans

Overall findings suggest that mobile van outlets of NSPs are effective in reaching hidden and high-risk group of IDUs. A large study (n = 1020) in Vancouver compared risk taking behaviours of IDUs attending conventional, and mobile van needle exchanges [46]. This demonstrated increasing risk profiles from IDUs who attend pharmacy, to those who attend conventional NSPs to mobile exchange van clients. Van users were more likely to be younger, Indigenous and female. These results are consistent with another Vancouver study [33], which compared van to conventional NSP users. Van users were found to inject more frequently, inject more frequently on the street, be younger, more likely to engage in sex work and less likely to be enrolled in a drug treatment program.

Riley et al. [36] studied new clients of both a mobile van and of a pharmacy-based NEP in the same neighbourhood in Baltimore. They found that the van attracted twice as many high-frequency injectors. Similarly, "Blue Bus" exchange in Lithuania reports successfully reaching a particular local community, identified as one of the most at risk groups, where injecting drug use is common. A survey conducted to evaluate the impact of the Blue Bus service on injecting practices of its clients revealed that within the previous 30 days 96% of IDUs reported they did not utilize used syringes, 88% did not share used syringes and needles with others, and 92% said they did not buy syringes already filled with a narcotic [50].

According to official data from the Ministry of Health, less than half the IDUs in Rome were in touch with drug dependence treatment units during 1992. However, with the aid of an outreach mobile van, 1023 (52.5%) new IDUs (who were not attending other services) were provided with services from the van in a one year period during 1992–93 [27]. Similarly, a mobile van outreach program in Spain encountered 1,745 new clients in only a 9 month period [29]. Comparable results have been reported on an evaluation of a pilot program using a camper van in Catania, Sicily. Although the camper van suffered a lack of active support from other drug treatment agencies and organisations, it slowly was able to establish contacts with an increasing number of hidden IDUs [38]. Lhomme et al. [21] reported findings of an evaluation of a NEP in Paris, which introduced mobile vans in its second phase. Of those accessing the program, 60% were homeless and 46% HIV positive of whom 59% were without medical follow-up.

### Complementary or duplication of services?

As evidence for the ability of these two types of NSP outlets to reach the hidden, hard-to-rich and high-risk group of IDUs has accumulated, it would be hoped that they complement each other and other modes of NSPs. Only two studies are available that have evaluated both dispensing machines and mobile vans. Agnoletto et al. [24] studied IDUs who used exchange machines and/or mobile vans but were not in contact with other health services in Milan, Italy. The authors concluded that the need to provide non-judgemental access to counselling and information justify complementary utilization of both exchange machines and mobile units as strategies for harm reduction. This observation is in keeping with the findings of a Berlin study that found users of vending machines, low-threshold meeting places and needle-exchange buses were significantly different in terms of HIV-rate, history of drug use and contact with counselling units [28]. Therefore they are different target groups for HIV-prevention. The most common three recommendations from IDUs (n = 76) interviewed in Scotland to improve access and quality of services were (i) outreach schemes and vending machines (62%), (ii) extending opening hours (12%) and (iii) more privacy in NEP (9%) [57]. These findings support the relevance of these two outlets in the context of other modes of NSP.

The most important advantage of dispensing machines is their anonymous and off-peak services when other outlets are closed. The findings of French trials consistently found that these machines are a useful adjunct to other modes of NSPs by reaching a different segment of IDUs [39,45]. All four evaluations [26,32,42,52] and a focus group discussion with IDUs [17] in Australia also supported the complementary role of dispensing machines.

Mobile vans mostly provide a flexible outreach service and act as a bridge to fixed-site outlets. For example, in Volgograd (Russian Federation) a fixed-site is augmented by a bus which serves three networks of drug users who live far apart in the city that stretches 40 km along the Volga river [58]. Similarly, Somlai et al. [34] described a mobile service, Lifepoint, which visits a number of high traffic areas on a rotating basis. These areas include drug houses, taverns, parks, and commercial sex areas. The duration of each visit to each site varies according to drug house locations, seasonal migrations of clients during cold weather, and in response to advice from key informants.

In some countries, amidst strong injecting paraphernalia law and few or no dispensing machine outlets on the grounds of importance of health staff contact, the mobile van can reduce the distance for users to travel to get needles and syringes. Carrying used syringes for a long periods in order to exchange presents problems for IDUs in the presence of police pressure and can dissuade them from bringing used syringes back [38]. The van reduces the risk of being caught by a law enforcing agency. Burrows [58] reminds us that forced closure is the most common reason for NSPs terminating services, and mobile services are often easier for local residents to cope with and can prevent or overcome the opposition that is focused on a fixed-site NSP. On the other hand, some IDUs in Vancouver mentioned the difficulty in meeting the mobile van as one of the major challenges [59], an issue vending machines may address effectively.

Importantly, while dispensing machines ensure great anonymity, they take away the important contact of IDUs with heath staff. In contrast, mobile vans ensure the contact but reduce anonymity.

## Discussion

This review offers evidence to support the notion that dispensing machines and mobile vans can accommodate different patterns of user, diversifying services to meet various needs. Drug use is not confined to a nine-to-five schedule. Practitioner-feedback from the only NSP outlet in Australia that is staffed 24 hours a day and 365 days a year indicates that close to half of the services are provided between 6 pm and 6 am [60]. Nevertheless in many parts of the world, even where NSPs are on a strong platform, there are few if any access points to sterile injecting equipment during these hours. In such a context, the need for a 24-hour service is gravely felt and syringe dispensing machines have emerged as a simple and very effective tool.

It is known that bringing users into contact with people who can support and promote appropriate behaviour change is an important aspect of contemporary NSPs [61]. This aspect has been valued so strongly in the NSPs of USA that it has probably worked against the introduction of dispensing machines [62]. However, it has also been recognised that because of the illicit nature of drug use, some users are reluctant to use services which bring them into contact with anyone, and even the relatively anonymous services provided by local pharmacies [63]. For those people a non-contact service was needed and dispensing machines to supply sterile equipment have emerged as an aid to them.

Syringe dispensing machines are likely to be highly cost effective, and the main saving is in staff costs. Clearly the staff costs would be substantial if a 24 hours staffed service is provided. Berg [26] found that machines could be highly self-supporting at lower prices for equipment, and be highly cost-effective even if the equipment was dispensed free-of-cost. However, this cost saving is based only on the cost of provision of sterile equipment and does not take into account the potentially greater health promotion impact via staff-user contact at staffed NSP outlets [64]. On the other hand, a mobile van service can be relatively expensive as, in addition to personnel cost, it involves the purchase and maintenance of a vehicle and fuel costs [58].

However, services through mobile vans can be provided at both locations and times that are compatible with the IDUs' lives. Mobile vans increase accessibility for clients who do not have a vehicle or money for transportation, and/or may be too drug-impaired to drive to the fixed-site. The importance of having a service close to IDUs was observed in a study in New York [65]. The authors found that 81% of IDUs who lived nearby typically used a NEP compared to 59% of those who lived further away. In multiple logistic regression analysis, those who lived nearby remained 3 times more likely (adjusted OR = 2.89; 95% CI 2.06–4.06) to use NEP, and were less likely to have engaged in receptive syringe sharing at their last injection (adjusted OR = 0.45, 95% CI 0.24–0.86). Therefore, locating NSP services in areas convenient to large numbers of IDUs may be critical for prevention of blood-borne virus infection. It might not always be possible to set up fixed-site NSP in all strategically important points because a range of variables needs to be addressed before attempting to set-up and then make it responsive to the need of IDUs. The mobile van can come to aid in resolving this problem.

Despite having conventional NSPs and pharmacies available, IDUs might experience several barriers in accessing sterile equipment. It was found that those who reported difficulty with accessing sterile needles were 3.5 times more likely to report needle sharing than were people without difficulty [66]. Table 2, we developed, describes some common barriers to NSP access, the majority of which were reported in a study in Sydney [67]. It also helps us understand the likely ability of dispensing machines and mobile vans to improve the accessibility and acceptability of NSPs to IDUs by addressing several barriers that IDUs encounter with the conventional NSPs and pharmacy outlets.

**Table 2:** Standard of good practice of dispensing machine and mobile van to address some common barriers experienced by IDUs in accessing sterile injecting equipment from conventional NSPs and pharmacy outlets.

**Some common barriers to access**	**Vending Machines' ability to address**	**Mobile Vans' ability to address**
Worried about being seen as an IDU	Very good	Moderate
Do not feel comfortable to visit NSPs	Very good	Moderate
Worried about being seen by parents/relatives	Moderate	Moderate
Hours not open when needed	Very good	Moderate
Pharmacies do not want to sell	Very good	Very good
Did not like attitudes of pharmacy-staff	Very good	Very good
Did not know about NSPs	Very good^α^	Very good^α^
Too far to travel NSPs or Pharmacies	Very good^α^	Very good^α^
Did not like location of NSPs or Pharmacies	Very good^α^	Very good ^α^
Too many police around NSPs	Very good	Very good
Not easy to travel to NSP and pharmacy	Very good ^α^	Very good ^α^
NSPs are too busy	Very good	Very good
NSP too close to a methadone clinic	Very good ^α^	Very good ^α^
Limited equipment available at once	Very good^β^	Very good ^δ^
Did not like NSP staff	Very good	Very good ^γ^
Did not understand the language	Very good	Moderate
NSPs are male dominated	Very good	Moderate ^Ω^

α: If strategically important places are covered by dispensing machines and (or) vans; β: If needle-syringes are offered for coin or free of cost; δ: If not strict to one-to-one exchange; γ: If peer staff are employed; Ω: It may be very good if services are gender responsive.

The results of this review do not support one type of NSP outlet over another, rather they suggest that coexistence of different modes and tailoring of services offered at different venues might be an important consideration. There is a convincing body of international experience on the effectiveness of conventional NSPs in providing access to sterile injecting equipment to IDUs, which in many settings cannot be replaced by other modes [68]. Nor on the other hand is it feasible to replace the advantages of a mobile van or dispensing machine by a conventional outlet. Cox et al. [69] recommends comprehensive NSPs including pharmacy involvement in distribution, strategically-placed dispensing machines and mobile exchanges.

This review should be considered in the light of several limitations. Firstly, only literature published in the English language was reviewed. Subsequent reviews should consider translating publications in other languages to capture a greater range of evidence. Secondly, most of the literature originated from developed countries and may not be generalizable to the conditions of developing countries where the vast majority of the world's IDUs now live. Thirdly, there is a paucity of data even in the grey literature. In addition, the articles reviewed may be subject to various biases.

## Conclusion

There is persuasive evidence that different venues of NSP attract different clients. In particular, dispensing machines and mobile vans are preferred modalities for hidden and high-risk IDUs. These two modalities can successfully address concerns about temporal and spatial accessibility and overall acceptability of NSP. Intrinsic advantages of each can offset the shortcomings of the other. Despite the relatively small volume of publications a clear and consistent finding is that these two outlets, if set up properly in a well chosen location with the local community well prepared, can generally increase the availability of sterile injecting equipment at times and places where coverage is poor. They also may enhance NSP provision through providing anonymous and confidential access to sterile injecting equipment for hidden and high-risk groups of IDUs.

## Statement of competing interests

The author(s) declare that they have no competing interests.

## Authors' contributions

MMI conceived of the review, collected the available background articles/reports on this topic and wrote the first draft of the manuscript. KMC critically analysed the manuscripts, corrected and revised all the versions. Both the authors read and approved the final manuscript.
